# Supplementary material for: The Construction and Exploration of a Comprehensive MicroRNA Centered Regulatory Network in Foxtail Millet (Setaria italica L.)
Source: Front Plant Sci. 2022 May 6;13:848474. doi: 10.3389/fpls.2022.848474 (PMC9121102; doi:10.3389/fpls.2022.848474)
Supplement: Supplementary file 2 [file Data_Sheet_2.zip › Sit-miR168_Seita.9G401700.1_269_TPlot.pdf]

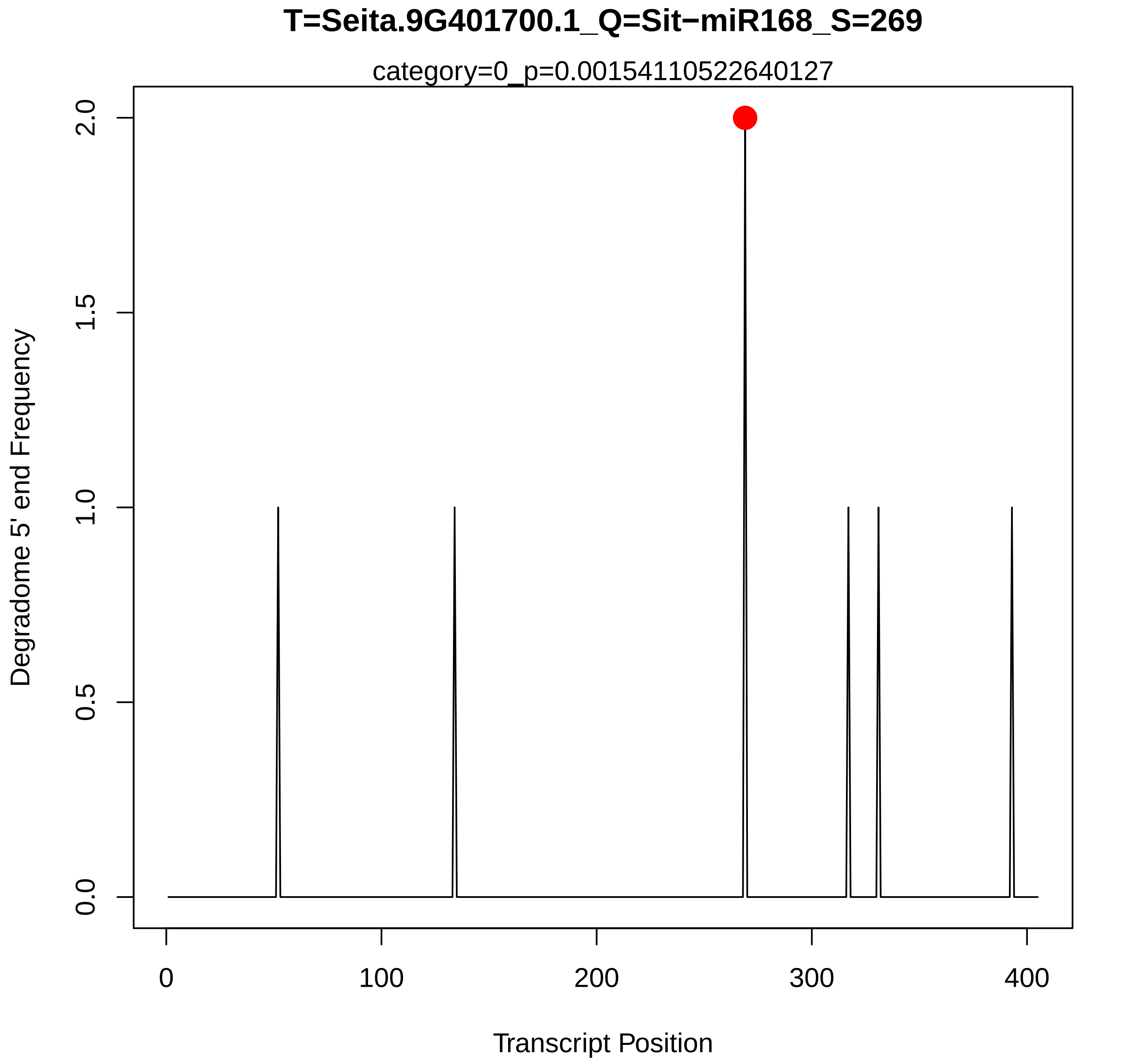

T=Seita.9G401700.1_Q=Sit−miR168_S=269
category=0_p=0.00154110522640127
Degradome 5' end Frequency
0.0
0.5
1.0
1.5
2.0
0
100
200
300
400
Transcript Position
